# Bioaerosol sampling of patients with suspected pulmonary tuberculosis: a study protocol

**DOI:** 10.1186/s12879-020-05278-y

**Published:** 2020-08-08

**Authors:** Benjamin Patterson, Anastasia Koch, Sophia Gessner, Ryan Dinkele, Melitta Gqada, Wayne Bryden, Frank Cobelens, Francesca Little, Digby F. Warner, Robin Wood

**Affiliations:** 1 University of Amsterdam, Amsterdam Institute for Global Health and Development, Amsterdam, the Netherlands; 2 Institute of Infectious Disease and Molecular Medicine (IDM), Faculty of Health Sciences, University of Cape Town, Cape Town, South Africa; 3 Desmond Tutu HIV Centre, Institute of Infectious Disease and Molecular Medicine (IDM), University of Cape Town, Cape Town, South Africa; 4 Zeteo Tech LLC, Ellicott City, MD USA; 5 Department of Statistical Sciences, University of Cape Town, Cape Town, South Africa

## Abstract

**Background:**

Tuberculosis (TB) is transmitted in bioaerosols containing *Mycobacterium tuberculosis* (*Mtb*). Despite being central to ongoing TB transmission, no routine diagnostic assay exists to measure *Mtb* in bioaerosols. Furthermore, published studies of *Mtb* in bioaerosol samples have been limited to individuals with sputum-positive pulmonary TB. Notably, TB diagnosis is based on clinical symptoms and sputum laboratory findings. This is despite the fact that approximately half of all patients commencing TB treatment are sputum-negative, resulting in a high proportion of presumptive treatments. Here, we propose to use a sensitive air sampling protocol to investigate the prevalence of *Mtb*-containing bioaerosols in both sputum-positive and sputum-negative TB suspects, at the same time evaluating the potential to identify unrecognized transmitters of TB.

**Methods:**

Our parallel-group design will identify viable *Mtb* in bioaerosols produced by individuals attending a TB clinic in South Africa. Sampling will be performed on eligible individuals presenting with symptoms indicative of TB and repeated at 14 days if initially positive. Participants will be prospectively classified into three distinct groups based on National TB Control Program (NTBCP) criteria: Group A, TB notification with sputum-based laboratory confirmation; Group B, TB notification with empiric diagnosis; and Group C, individuals not notified. Group C individuals with detectable *Mtb* bioaerosol will be monitored until resolution of clinical and laboratory status. Collection of bioaerosol specimens will be via two consecutive sampling modalities: (1) direct sampling following a specific respiratory manoeuvre; and (2) indirect sampling during passive respiratory activity. Bioaerosol specimens will be analyzed for viable *Mtb* using DMN-trehalose staining and live-cell fluorescence microscopy. *Mtb* genomes and mycobacterial and host lipids will be detected using droplet digital PCR and mass spectrometry analyses, respectively. The primary objective is to determine the prevalence of *Mtb* bioaerosols in all TB clinic attendees and in each of the groups. Secondary objectives are to investigate differences in prevalence of *Mtb* bioaerosol by HIV status and current isoniazid preventive therapy (IPT) use; we will also determine the impact of anti-TB chemotherapy on *Mtb*-containing bioaerosol production.

**Discussion:**

Respiratory bioaerosol has a potential role in non-invasive TB diagnosis, infectivity measurement and treatment monitoring.

**Trial registration:**

ClinicalTrials.gov: NCT04241809. Date of Registration: 27/1/2020.

## Background

Tuberculosis (TB), which is caused by the obligate human pathogen, *Mycobacterium tuberculosis* (*Mtb*), is the foremost infectious cause of death in adults globally [1]. Persistence of the TB epidemic is driven by successive cycles of infection, pulmonary disease, *Mtb* bioaerosol expulsion and inhalation by susceptible individuals. Interventions to interrupt this cycle are critical to curb the TB epidemic, however knowledge of the physical process of *Mtb* transmission is limited. This is primarily due to the paucity of investigations detecting, quantifying and characterizing *Mtb* bioaerosols. As others have highlighted [2], it remains unclear when source cases become infectious, how long after effective treatment they remain infectious, what are the respiratory mechanisms of aerosolization, and how long *Mtb* bioaerosols remain viable in the environment. Furthermore, while sputum *Mtb* positivity and presence of lung cavitation predict infectiousness, prediction is partial (and based on retrospective, epidemiological analyses) thus limiting accurate risk stratification for contact screening. To address these knowledge gaps, tools to facilitate direct detection of viable aerosolized *Mtb* bacilli in exhaled breath are urgently required.

To improve the utility of *Mtb* bioaerosol sampling, it is essential to maximize detection yield. Three key elements to consider are: (1) patient bioaerosol production; (2) an efficient bioaerosol collection apparatus; and (3) the *Mtb* detection modality. Previous bioaerosol studies have used a spectrum of sampling approaches. These include subjects coughing into an apparatus for 10 min with culture-based detection [3], sitting passively in a chamber for 1 h with a combination of culture and PCR detection [4], and wearing a modified face mask for 10 min to 5 h with PCR detection [5, 6]. Though instructive, these studies have been limited to individuals with sputum-positive pulmonary TB, with detection yields ranging from 28 to 77%. This range of outcomes can be attributed to the spectrum of inter-individual infectiousness; however, the unknown variance in intrinsic sensitivity of each of the systems is likely to be more significant. Put simply, the absence of *Mtb* bioaerosol in a sampled individual may only indicate *Mtb* numbers below the detection threshold of the sampling system, rather than non-production. Consequently, optimizing all elements of the sampling process is essential to identify *Mtb* bioaerosol in paucibacillary individuals. Critical factors include the site in the respiratory tract of bioaerosol generation [7], bioaerosol volume obtained, and the limit of detection of the technique used to identify the organism. Bioaerosol specimen volumes are likely to be in the order of nanolitres so, even with *Mtb* bacilli concentrations greater than in sputum [4], standard assays may be inadequate.

This study will assess an optimized bioaerosol sampling system based on the previously described Respiratory Aerosol Sampling Chamber (RASC) [8]. Patient-derived bioaerosol generated by specific respiratory manoeuvres will be directly captured to maximize aerosol volume. In addition, passive respiratory activity within a chamber with indirect air sampling will be used to capture bioaerosol with transmission potential. A high-flow, wet-walled cyclone collector will be used for both methods, ensuring the capture of a large proportion of the exhaled air volumes with extraction of the bioaerosol into liquid. The liquid specimen will then be subjected to live-cell fluorescence imaging to identify single viable *Mtb* bacilli based on DMN-trehalose probe incorporation patterns and differential bacterial cellular morphology [9].

### Aim

The aim of this study is to identify viable *Mtb* bacilli in exhaled bioaerosol from individuals attending a TB clinic in Cape Town, South Africa. The specific objectives are divided into primary, secondary and exploratory.

#### Primary objective

1. To compare the prevalence proportions of *Mtb*-containing bioaerosol in sputum-positive and sputum-negative pulmonary TB cases (Groups A & B).

#### Secondary objectives

2. To quantify the numbers of viable *Mtb* organisms in bioaerosols per litre of air sampled and per nanolitre of bioaerosol particulate volume collected in both sputum-positive and sputum-negative pulmonary TB cases (Groups A & B).

#### Exploratory objectives

3. To determine the prevalence of *Mtb*-containing bioaerosols in TB suspects not diagnosed with clinical TB (Group C).
4. To determine the prevalence and numbers of viable *Mtb* in subjects receiving or not receiving IPT prophylaxis.
5. To determine the prevalence and numbers of viable *Mtb* in subjects with and without HIV co-infection.
6. To determine the prevalence and numbers of viable *Mtb* in subjects before and after TB therapy.
7. To determine the numbers of viable *Mtb* in bioaerosols collected by direct and indirect sampling.
8. To determine the ratio of *Mtb* genomes (ddPCR) to viable *Mtb* organisms (DMN-tre positivity) in *Mtb*-containing bioaerosols.
9. To identify which *Mtb* and host lipids are present in *Mtb*-containing bioaerosols.

## Methods

### Study design

**Fig. 1:**
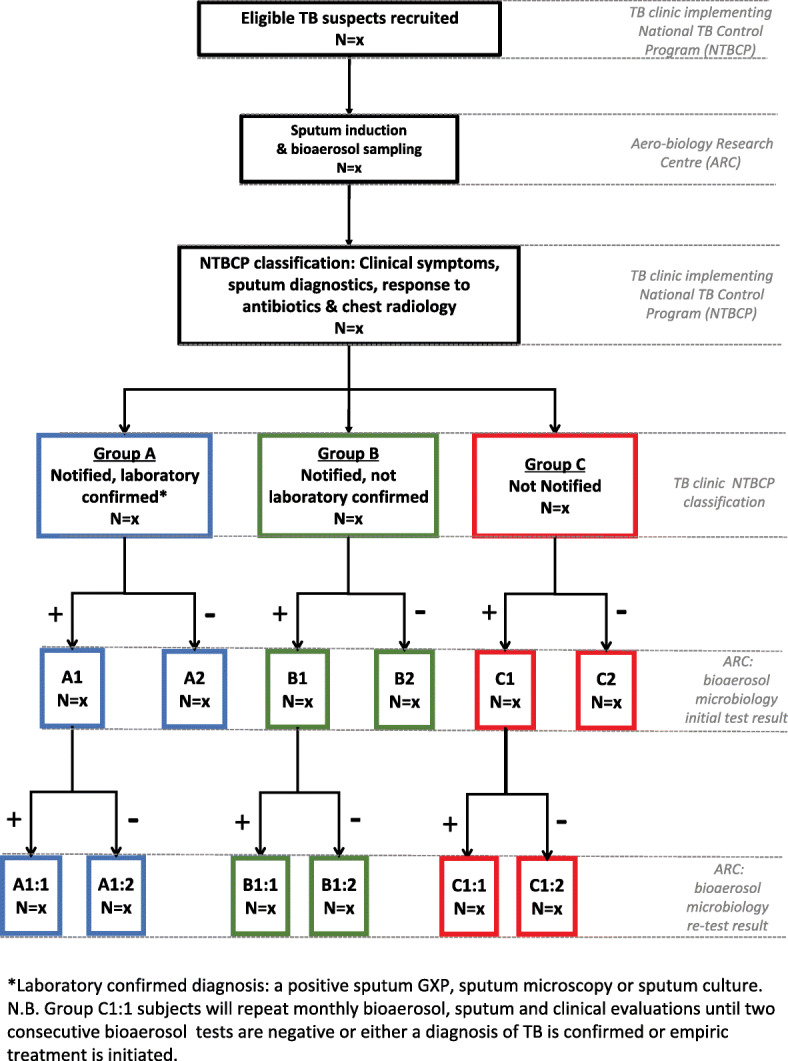
Study flow diagram

This study will utilize a pragmatic, parallel group design (Fig. 1). All participants who meet the eligibility criteria will be invited to have bioaerosol sampling performed at the Aerobiology Research Centre (ARC) prior to TB investigations or treatment. An induced sputum sample will be collected, cultured and stored for future downstream studies before bioaerosol sampling. Classification into three groups will be based on the outcome of routine clinical practice at the TB clinic implementing the National TB Control Program (NTBCP) [10]. Group A will comprise patients with TB notification and sputum-based laboratory confirmation (GeneXpert positive sputum or sputum culture if performed); Group B will consist of patients with TB notification without sputum-based laboratory confirmation (physician diagnosed cases with GeneXpert negative sputum and negative sputum culture if performed); and Group C will contain individuals who are not notified. Repeat bioaerosol sampling will be conducted after 14 days on participants in all groups with initial bioaerosol samples containing viable *Mtb*. Group C individuals will be re-evaluated clinically, with induced sputa and repeated bioaerosol sampling at monthly intervals until two consecutive samples are negative or treatment is commenced.

### Study population

Consecutive patients with respiratory and/or constitutional symptoms suggestive of pulmonary TB who self-present to either of two local TB clinics will be offered recruitment into the study. The expected timeline to recruit the target number of patients is 1–2 years.

The eligibility criteria are:

- age over 13 years (corresponding to increasing TB prevalence)
- suspected TB
- written, informed consent (parental consent and patient assent for those aged under 18 years)

### Bioaerosol collection

Bioaerosol specimens will be collected by two sampling methods. In addition, exhaled breath will be simultaneously sampled for aerodynamic particle counting and the CO_2_ concentration of sampled air will be measured. Ozone sterilization and empty RASC sampling will be carried out between every participant to provide negative controls.

### Direct sampling

**Fig. 2:**
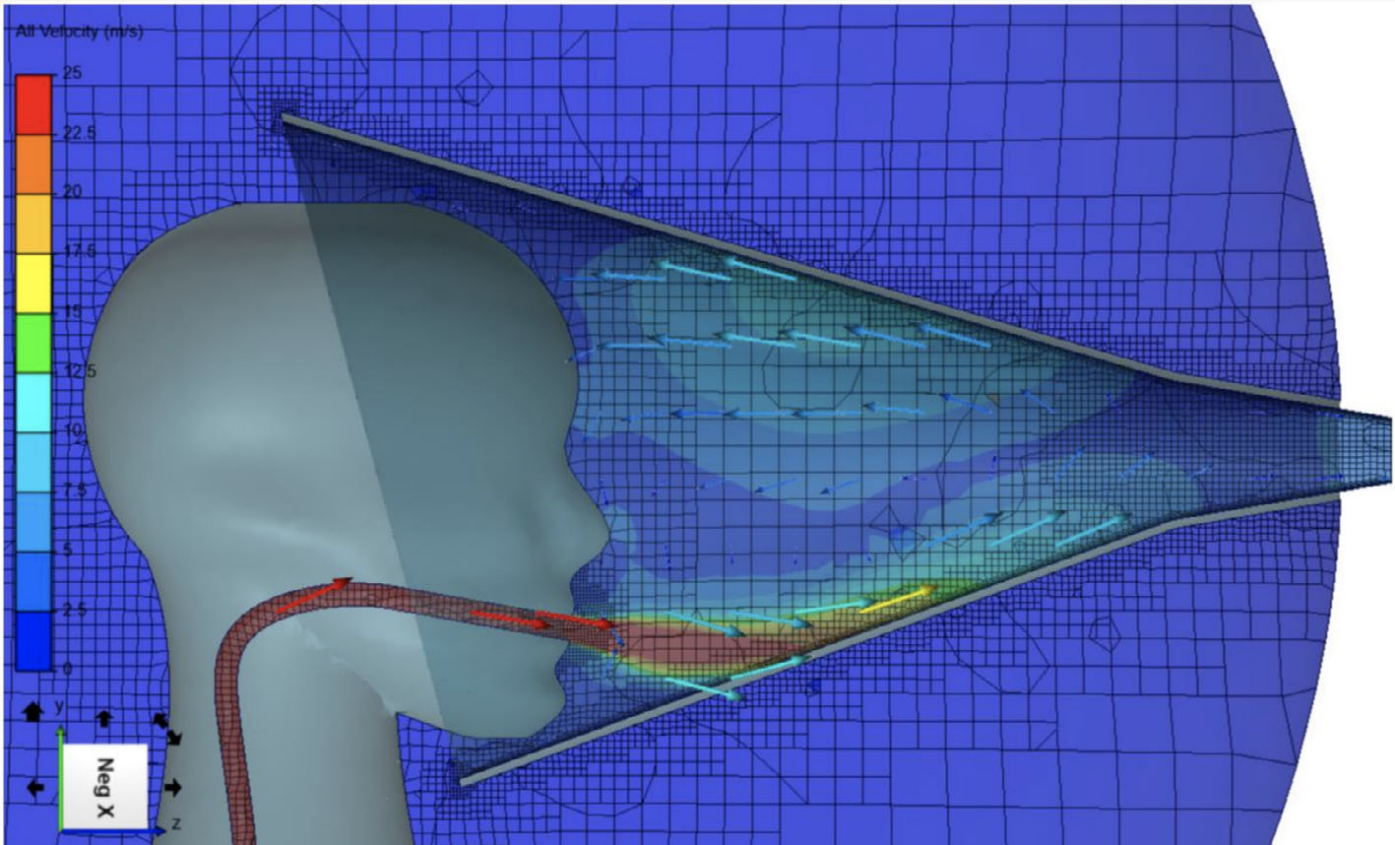
Modified RASC with author wearing a Tyvek™ non-woven fabric suit

**Fig. 3:**
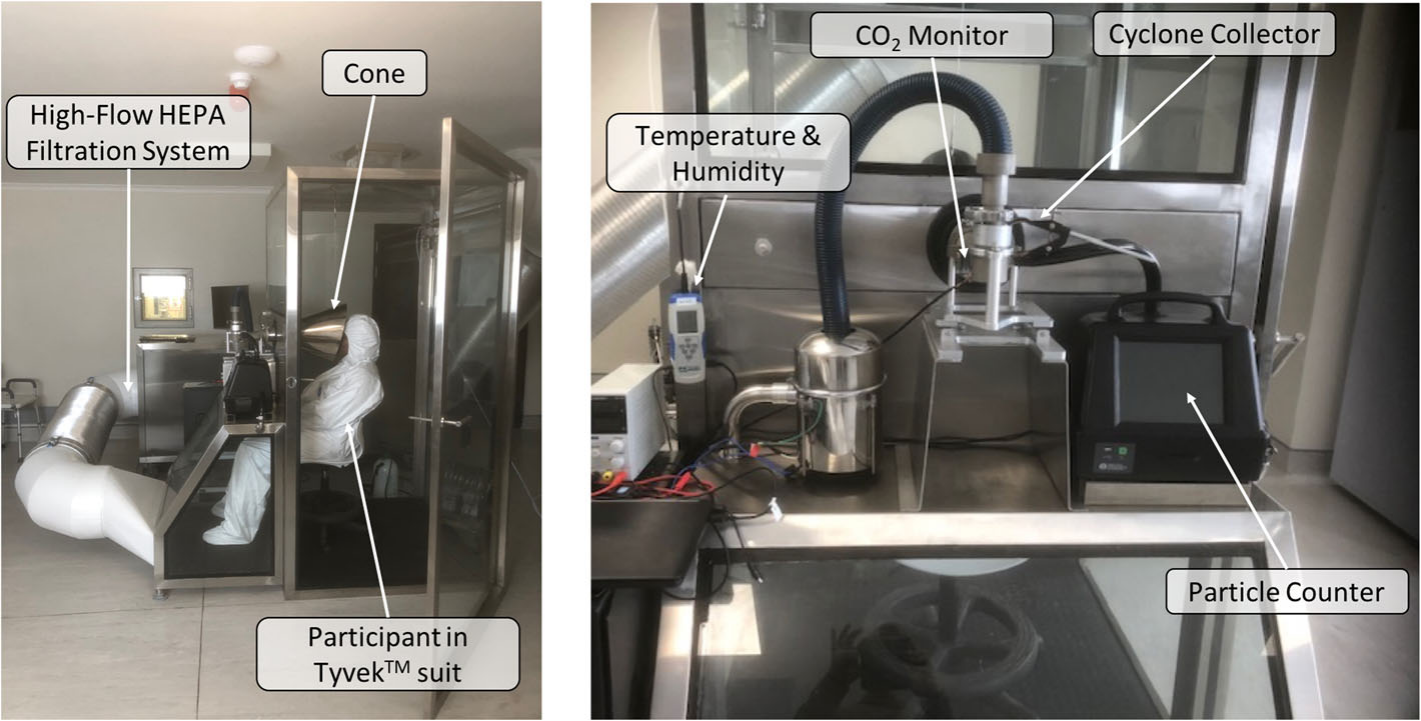
Computational fluid dynamic modelling of the flow velocity streams at maximum cough strength with a collection air-flow rate of 300 l per minute

A direct sampling protocol was developed by modifying the RASC [8]. Briefly, the RASC is a micro-environment designed for investigation of respiratory bioaerosol emissions from a single individual (see Fig. 2). This small personal clean space accommodates a seated participant during sampling. Modified from earlier descriptions, a metallic elliptical cone has been incorporated into which the participant can comfortably place their head at the larger open end. A unidirectional airflow is created by connection of a high-flow (250 l per minute) bespoke cyclone collector connected at the cone apex which extracts bioaerosol into a sterile phosphate-buffered solution. Air enters the collector via a tangential nozzle which generates a liquid cyclone with particle inertia leading to deposition of bioaerosol from the airstream onto the wet wall. The exit airflow from the cone will reaches a velocity of 12.5 m per second, enabling full collection of all expiratory respiratory particles including those generated during forceful coughing. Participants, who will be seated in the HEPA-filtered chamber, will be required to perform a specific breathing protocol with regular instruction by a study nurse. The protocol will consist of a series of 15 vigorous coughs every 20 s (5 mins), a period of tidal breathing (5 mins) and a further series of 15 bronchiole burst manoeuvres (BBM) every 20 s (5 mins). A BBM consists of two deep breaths with full exhalation on each breath. Figure 3 illustrates computational fluid dynamic modelling demonstrating complete sampling of high-velocity respiratory manoeuvres without backflow from the cone. Collection of entrained bioaerosol from the respiratory activities to a small volume of liquid enables non-culture-based analytical methods as described below [7].

### Indirect sampling

Sampling of patient-derived respiratory bioaerosol during natural respiratory activity will also be performed in the same chamber without the use of the elliptical cone. This method is similar to a previous study [4] but has since been adapted to improve sensitivity [11]. In order to replicate a real-life transmission environment the entire volume of the RASC concentrates exhaled air leading to a variable dwell period with particle ageing prior to collection. This sampling takes place over a 60-min period following the direct sampling protocol. The high-flow cyclone collector continuously samples chamber air extracting bioaerosol into a sterile phosphate-buffered saline solution.

### Microscopy-based detection of *Mtb*

The pellet from the centrifuged bioaerosol sample is stained using the solvatochromic trehalose probe, DMN-trehalose, which is incorporated as trehalose monomycolate into the cell walls of metabolically active *Corynebacteriaceae* (including *Mycobacterium*) by the antigen 85 protein complex [12]. After overnight incubation in media containing DMN-trehalose, the samples are washed and added to a novel nanowell device described in ref. [9]. The devices are sealed with adhesive film and centrifuged prior to visualization by fluorescence microscopy. Detection, enumeration and differentiation from debris of fluorescent bacilli is based on dye incorporation patterns and differential bacterial cellular morphology (cell length and width) [9].

### Droplet digital polymerase chain reaction (ddPCR) detection of *Mtb*

DNA from *Mtb* bacilli is extracted using an in-house lysis buffer with subsequent pelleting of DNA (by centrifugation at 9000 x g for 10 min), discarding of supernatant, and resuspension in 20 μl of Tris-EDTA buffer (10 mM Tris, 1 mM EDTA, pH 8.0). Given that ddPCR is relatively robust against inhibitors, no further DNA purification is required. The primer/probe combinations and reaction conditions for *Mtb* specific ddPCR have been described previously [4]. Serial dilutions of known concentrations of purified *Mtb* H37Rv genomic DNA are included as positive internal controls for ddPCR, and nuclease-free water is included as a negative control. Data generated from the ddPCR reaction are analyzed via the Umbrella pipeline [13] using only wells for which a minimum of 10,000 droplets have been detected.

### Mass spectrometry detection of *Mtb*

The supernatant from the centrifuged bioaerosol sample is lyophilized followed by lipid extraction by the Folch method. Samples are introduced into an LTQ Orbitrap Mass Spectrometer (Thermo Fisher Scientific) via electrospray ionization by direct infusion. Positive and negative ion calibration solutions are used to optimize ion source conditions and mass calibration. For data acquisition, resolving power of 60,000 at 200 m/z is used for both positive and negative ions in the mass range 200–2000 m/z.

### Statistical analysis

Historical clinic data suggest individuals suspected of TB divide into groups A, B, and C in the ratio 3:5:10. Based on this ratio, a sample size of 250 TB clinic attendees will give a 90% power to detect a 20% bioaerosol yield in Group B. Assuming 100% bioaerosol yield in Group A, this gives an absolute difference of 12.5% between sputum and bioaerosol yields in notified TB cases (two-sided *p* < 0.05).

Demographic (age, sex, BMI, HIV status) and microbiology data will be entered into a secure RedCap Database. To maintain confidentiality, participant identifying information will be removed. All analyses will be based on the groups to which patients are originally assigned. Analyses of the primary and secondary aims will be by comparison of paired proportions using McNemar’s test or comparison of independent proportions using a chi-square test. Mixed effect models can be used to adjust comparisons for any potential confounding variables.

### Ethics statement

Ethical approval has been obtained from the University of Cape Town Faculty of Health Sciences Human Research Ethics Committee (HREC/REF: 529/2019). Written informed consent including for publication of medical details will be obtained from the participants, with parental consent and participant assent for those under 18 years of age.

## Discussion

The implications of bioaerosol positivity in people with suspected pulmonary TB differ depending on the subgroup sampled. Prevalence in patients with sputum-confirmed TB (Group A) establishes the sensitivity of bioaerosol sampling comparable with previous studies [3–5]. This will validate the optimized bioaerosol sampling system and allow a comparison between the direct and indirect aerosol generation methods. The direct method – performing a respiratory manoeuvre into a cone apparatus – captures nascent bioaerosol from the lung periphery and upper airway structures. The short time-interval between mouth and collector (approx. 40 msec) reduces losses of large particles by gravitational settling and maximizes the bioaerosol volume captured. Therefore, this approach aims to capture all *Mtb* organisms produced from the respiratory tract regardless of potential role in transmission. In contrast, the indirect capture method samples an aged bioaerosol product from the close breathing zone of the patient. The smaller size distribution of bioaerosol following drop-out of the larger diameters and desiccation to hygroscopic equilibrium has the capacity to penetrate the lungs of susceptible contacts. It follows, therefore, that this bioaerosol has the greatest propensity for disease transmission. Furthermore, enumeration of viable *Mtb* bacilli derived from this method may more closely reflect host infectivity, as similar investigations have related high numbers of *Mtb* colony forming units in cough aerosols to increased infection rates in household contacts [14]. Using the indirect method, we intend to explore the effect of treatment on organism numbers longitudinally by repeat sampling of patients with bioaerosol positivity in groups A and B. Also, by identifying the subset of HIV-infected individuals already receiving IPT at enrolment we will be able to assess the effect of isoniazid monotherapy on *Mtb* bioaerosol production.

To our knowledge, bioaerosol sampling in sputum-negative pulmonary TB cases has not been performed previously, despite evidence of transmission from this group [15–18]. We are therefore extending attempts to detect bioaerosol to patients with notified TB without sputum microbiological confirmation (Group B). Expectorated sputum, the primary specimen for pulmonary TB diagnosis, has significant limitations [19]. Globally, only 56% of pulmonary TB cases are bacteriologically confirmed by sputum testing [20]. The remainder, who are diagnosed by clinical symptoms or chest radiography, do not accumulate *Mtb* bacilli in sputum, or do not produce sputum, or do not expectorate adequately. Poor quality sputum samples are a particular problem in children and in HIV-infected individuals, leading to suboptimal diagnostic sensitivity. The finding of culture or Xpert MTB/RIF positivity in bronchoalveolar lavage from sputum-negative or sputum-scarce patients [21–23] suggests that *Mtb* is frequently present in the peripheral airways. The bronchiole fluid film burst method of bioaerosol generation [7] rapidly and non-invasively samples this biofluid with comparable biosafety to induced sputum. We aim to provide preliminary data on the clinically relevant role of bioaerosol sampling as a novel diagnostic specimen.

Identifying *Mtb* in bioaerosols from patients investigated but not treated for TB (Group C) indicates either a missed diagnosis or *Mtb* transmitters in the absence of discernible TB disease. Observations from multiple studies in different high-burden settings indicate that only 1–30% of new *Mtb* infections can be linked with known TB cases [24–27]. This is consistent with the existence of many unrecognized transmitters in TB endemic communities. We hypothesize that asymptomatic or minimally symptomatic individuals represent a major driver of transmission. However, it has been historically difficult to investigate the infectiousness of this subpopulation, partially owing to the dependence on sputum for diagnosis. Our intention to sample bioaerosols from individuals suspected of TB prior to investigation will opportunistically include many individuals not ultimately diagnosed with TB. Although these individuals will not be asymptomatic, they will nonetheless be *Mtb* transmitters who are not on treatment. As an exploratory aim, individuals identified with bioaerosol positivity will be re-sampled at weekly intervals (in addition to thorough clinical re-evaluation) to characterize the natural history of sub-clinical *Mtb* shedding.

This study aims to demonstrate the utility of bioaerosol sampling for TB diagnosis and therapeutic monitoring. High rates of successful bioaerosol *Mtb* detection across groups of TB clinic attendees would support the future development of bioaerosol sampling methods to inform TB control strategies. Widespread application may potentially reduce diagnostic uncertainty and enable improved case-finding with both individual and public health benefit.

### Dissemination

The outcomes of the analysis in addition to the unprocessed data will be submitted for publication in open-source peer-reviewed journals and presented at international conferences.

## Data Availability

Not applicable.

## Abbreviations

*Mtb*: *Mycobacterium tuberculosis*
TB: Tuberculosis
RASC: Respiratory Aerosol Sampling Chamber
ARC: Aerobiology Research Centre
NTBCP: National TB Control Program
PCR: polymerase chain reaction
