# Correction to: Bioaerosol sampling of patients with suspected pulmonary tuberculosis: a study protocol

**DOI:** 10.1186/s12879-020-05342-7

**Published:** 2020-08-24

**Authors:** Benjamin Patterson, Anastasia Koch, Sophia Gessner, Ryan Dinkele, Melitta Gqada, Wayne Bryden, Frank Cobelens, Francesca Little, Digby F. Warner, Robin Wood

**Affiliations:** 1 University of Amsterdam, Amsterdam Institute for Global Health and Development, Amsterdam, the Netherlands; 2 Institute of Infectious Disease and Molecular Medicine (IDM), Faculty of Health Sciences, University of Cape Town, Cape Town, South Africa; 3 Desmond Tutu HIV Centre, Institute of Infectious Disease and Molecular Medicine (IDM), University of Cape Town, Cape Town, South Africa; 4 Zeteo Tech LLC, Ellicott City, MD USA; 5 Department of Statistical Sciences, University of Cape Town, Cape Town, South Africa

**Correction to: BMC Infect Dis 20, 587 (2020)**

**https://doi.org/10.1186/s12879-020-05278-y**

Following publication of the original article [1], we were notified of a few errors in how the author corrections have been implemented:

- The images for Figs. 2 and 3 were reversed. The captions and references in the text were otherwise correct.
- Reference 20 should have been removed and the text refer to Reference 1 instead.
  The original article has been corrected.

**Fig. 2:**
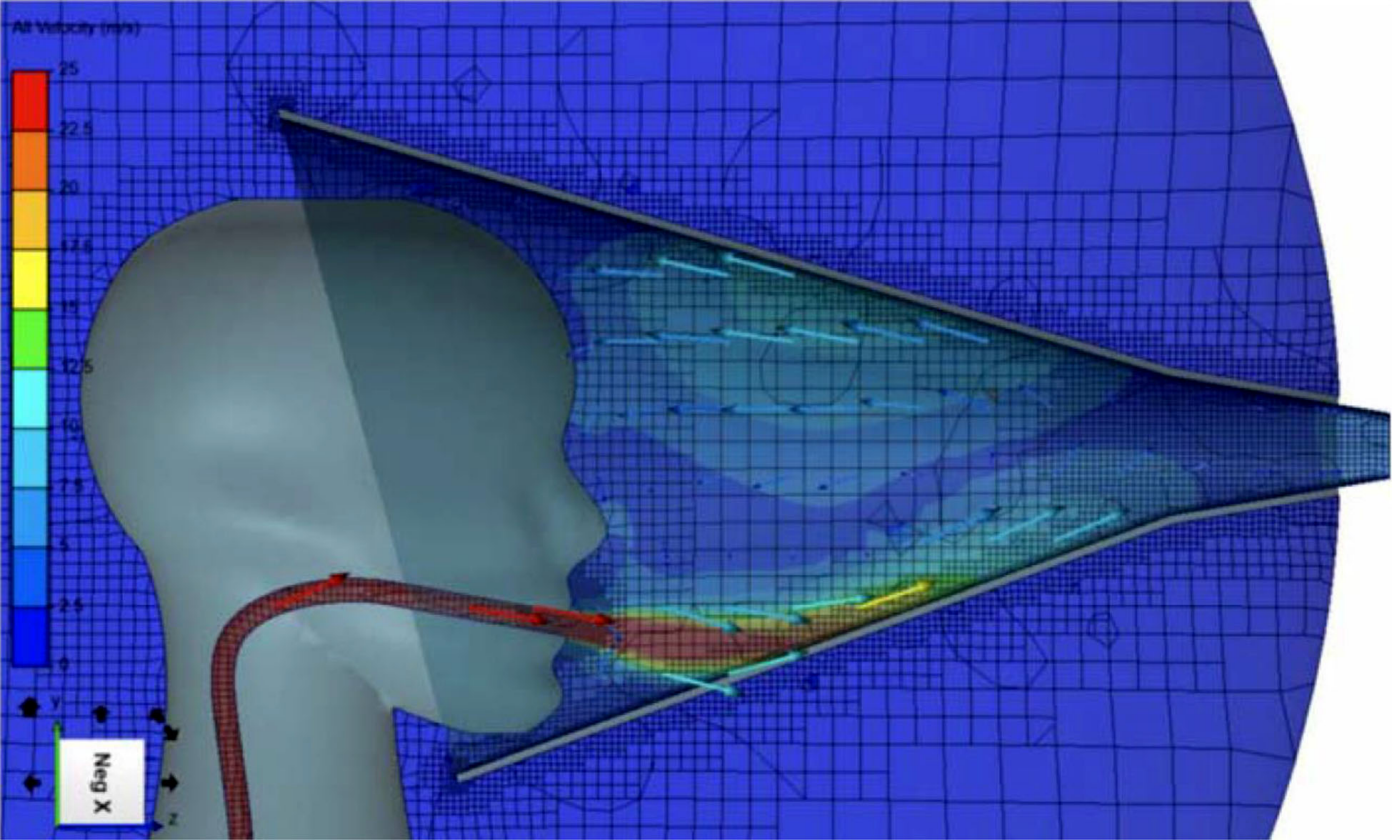
Computational fluid dynamic modelling of the flow velocity streams at maximum cough strength with a collection air-flow rate of 300 l per minute

**Fig. 3:**
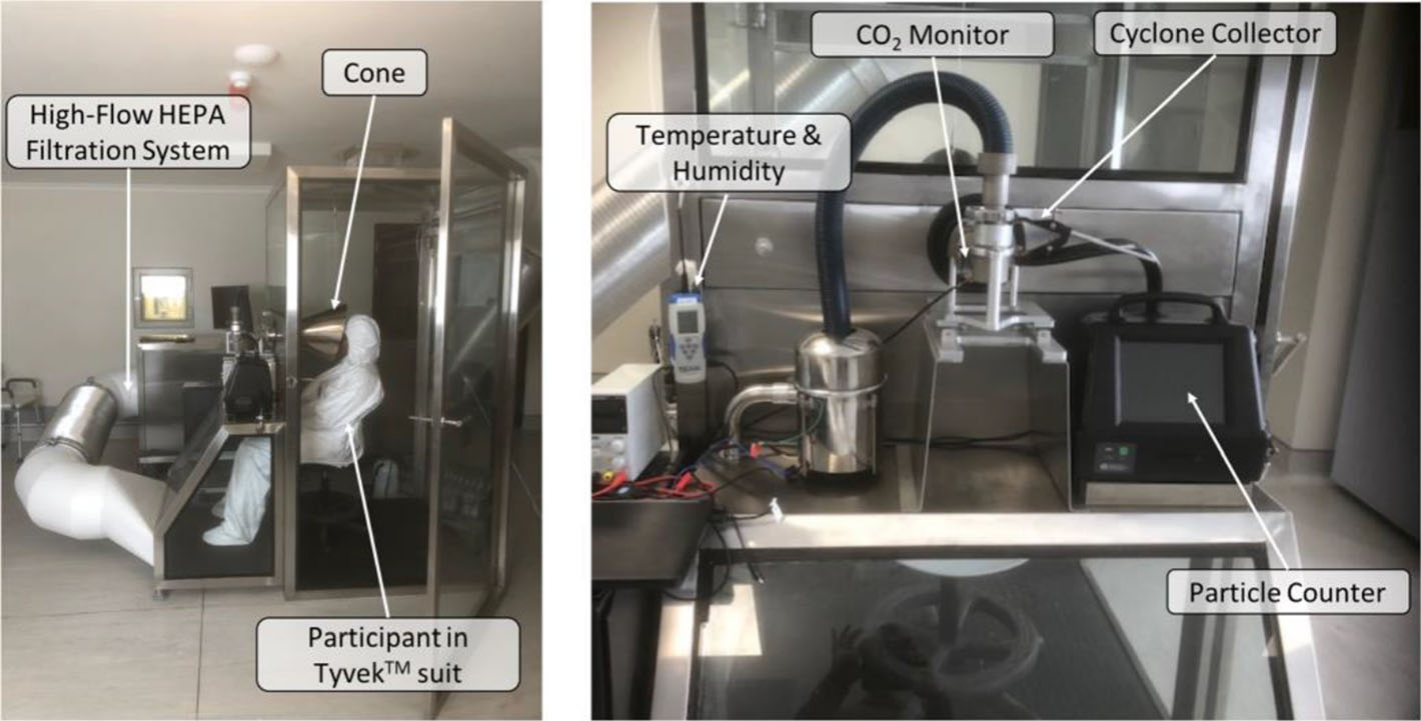
Modified RASC with author wearing a Tyvek™ non-woven fabric suit
